# Mechanical thrombectomy in intermediate- and high-risk acute pulmonary embolism: hemodynamic outcomes at three months

**DOI:** 10.1186/s12931-023-02552-w

**Published:** 2023-10-25

**Authors:** Lucas Lauder, Patricia Pérez Navarro, Felix Götzinger, Sebastian Ewen, Hussam Al Ghorani, Bernhard Haring, Philipp M. Lepper, Saarraaken Kulenthiran, Michael Böhm, Andreas Link, Bruno Scheller, Felix Mahfoud

**Affiliations:** 1 Klinik für Innere Medizin III – Kardiologie, Angiologie und Internistische Intensivmedizin, Universitätskliniken des Saarlandes und Universität des Saarlandes, Homburg, Germany; 2 Klinik für Innere Medizin V – Pneumologie, Allergologie und Intensivmedizin, Universitätskliniken des Saarlandes und Universität des Saarlandes, Homburg, Germany

**Keywords:** Pulmonary embolism, Mechanical thrombectomy

## Abstract

**Background:**

Mechanical thrombectomy has been shown to reduce thrombus burden and pulmonary artery pressure (PAP) and to improve right ventricular (RV) function in patients with high-risk or intermediate-high-risk pulmonary embolism (PE). As hemodynamic data after mechanical thrombectomy for PE are scarce, we aimed to assess the hemodynamic effects of mechanical thrombectomy in acute PE with right heart overload.

**Methods:**

In this prospective, open-label study, patients with acute symptomatic, computed tomography-documented PE with signs of right heart overload underwent mechanical thrombectomy using the FlowTriever System. Right heart catheterization was performed immediately before and after thrombectomy and after three months. Transthoracic echocardiography was performed before thrombectomy, discharge, and at three months. This analysis was done after 20 patients completed three months of follow-up.

**Results:**

Twenty-nine patients (34% female) underwent mechanical thrombectomy, of which 20 completed three months follow-up with right heart catheterization. Most patients were at high (17%) or intermediate-high (76%) risk and had bilateral PE (79%). Before thrombectomy, systolic PAP (sPAP) was severely elevated (mean 51.3 ± 11.6 mmHg). Mean sPAP dropped by -15.0 mmHg (95% confidence interval [CI]: -18.9 to -11.0; p < 0.001) immediately after the procedure and continued to decrease from post-thrombectomy to three months (-6.4 mmHg, 95% CI: -10-0 to -2.9; p = 0.002). RV/left ventricular (LV) ratio immediately reduced within two days by -0.37 (95% CI: -0.47 to -0.27; p < 0.001). The proportion of patients with a tricuspid annular plane systolic excursion (TAPSE)/sPAP ratio < 0.31 mm/mmHg decreased from 28% at baseline to 0% before discharge and at three months (p = 0.007). There were no procedure-related major adverse events.

**Conclusions:**

Mechanical thrombectomy for acute PE was safe and immediately reduced PAP and improved right heart function. The reduction in PAP was maintained at three months follow-up.

## Background

Pulmonary embolism (PE) remains the leading cause of preventable death in hospitalised patients [1, 2]. The 2019 European Society of Cardiology (ESC) Guidelines recommend a risk-adjusted approach for the management of acute PE by stratifying the risk of early death based on clinical and hemodynamic parameters [3]. In low- and intermediate-low-risk patients, treatment consists of therapeutic anticoagulation [3]. Patients with high-risk PE appear to benefit from reperfusion therapy, i.e. systemic thrombolysis [3]. However, systemic thrombolysis is underused, associated with bleeding complications, occasionally fails, [4–6] and is contraindicated in a subset of patients [5]. Additionally, patients with intermediate-high-risk PE do not benefit from systemic thrombolysis as bleeding complications often outweigh potential hemodynamic improvements [4, 7].

Whenever systemic lysis fails or is contraindicated, catheter-directed reperfusion treatment should be considered in patients with high-risk PE [3, 8–10]. In the FlowTriever Pulmonary Embolectomy (FLASH) study [11] and a large registry, [12] transcatheter thrombus aspiration using the FlowTriever System (Inari Medical, Irvine, CA) was shown to be feasible and safe in intermediate and high-risk PE. Moreover, mechanical thrombectomy reduced pulmonary artery pressures (PAP) and right ventricle (RV)/left ventricle (LV) ratio acutely after the procedure. However, whether the observed hemodynamic improvements following thrombectomy are maintained through longer-term follow-up is unknown.

Herein, we assessed the safety and the hemodynamic effects of mechanical thrombectomy in acute PE with right heart overload through three months of follow-up.

## Methods

### Study design and patient selection

This prospective, open-label, single-arm, single-center study assessed the hemodynamic effects of transcatheter mechanical thrombectomy using the FlowTriever System in acute symptomatic PE with right heart overload. This analysis was done after the first 20 patients completed their three months follow-up. Adults (≥ 18 years) with symptoms and clinical signs of acute (symptom duration < 30 days) PE, computed tomography-documented proximal filling defects in at least one main or lobar pulmonary artery, and signs of right heart overload in transthoracic echocardiography or computed tomography were included. Exclusion criteria were inability to receive anticoagulation and life expectancy < 30 days (as determined by the investigator). All patients provided written informed consent. The trial was approved by the local ethics committee (ethic committee of the Ärztekammer des Saarlandes) and complied with the Declaration of Helsinki.

### Procedures

All patients had computed tomography to confirm the diagnosis of pulmonary embolism. Transthoracic echocardiography was performed before the thrombectomy.

Mechanical thrombectomy was achieved using the FlowTriever System via large-bore transfemoral venous access. Full details of the procedure have been reported previously [11]. In brief, the Triever Catheter (16, 20, or 24 Fr) was advanced over an 0.035” guidewire into the pulmonary arteries for thrombus aspiration. If needed, the FlowTriever Catheter was advanced through the Triever Catheter over the guidewire for mechanical thrombus dislodgment and removal. Pulmonary angiography and right heart catheterization were performed immediately before and after the thrombectomy. A suture-based closure device was used. Procedure time was defined as the time from gaining venous access until sheath removal.

Right heart catheterization and transthoracic echocardiography were repeated at three months of follow-up.

### Outcomes

The primary safety outcomes were the number of patients with major adverse events, including major bleeding and periprocedural device- or procedure-related adverse events, between baseline to 48 h and survival at 30 days of follow-up. The efficacy outcomes were changes in sPAP between baseline and three months and changes in RV/LV ratio determined by transthoracic echocardiography from baseline to 48 h. Other outcomes included changes in echocardiographic parameters (tricuspid annular plane systolic excursion (TAPSE), TAPSE/sPAP ratio, and LV ejection fraction), and biomarkers, such as high-sensitivity (hs) troponin T and N-terminal B-type natriuretic peptide (NT-proBNP), as well as lengths of in-hospital stay, and time spent at intensive care unit (ICU).

### Statistical analysis

Statistical analyses were done using Stata 16.1 (StataCorp, College Station, TX, USA). Categorical variables are summarized as counts (percentages). Continuous variables are presented as means (standard deviations [SD]) or median (interquartile range [IQR]). The paired t-test or the Wilcoxon signed-ranks test was used to analyze matched pairs. Before-after changes are summarized as means (95% confidence intervals [CI]). McNemar’s (two paired samples) or Cochran’s Q test (more than two paired samples) were used to compare the proportion of patients with mean PAP (mPAP) > 20 mmHg within groups. Pearson’s correlation coefficient was calculated for systolic PAP before thrombectomy and its change during follow-up. All tests were two-sided, and p-values < 0.05 were considered significant.

## Results

From April 2021 to August 2022, 29 patients (34% female) were included. The patients’ baseline characteristics are summarized in Table 1. Most patients were at high (17%) or intermediate-high (76%) risk, according to the 2019 ESC acute PE Guidelines [3]. The mean PE severity index (PESI) [13] was 121 ± 38. Bilateral PE occurred in 79% of the patients. The median time from the symptom onset to the procedure was 21 h (IQR: 4–24). The median procedural time was 68 min (IQR: 60–90). The procedural characteristics are summarized in Table 2. The median ICU and in-hospital length of stay were 2 days (IQR: 1–4) and 7 days (IQR: 6–11), respectively. At discharge, 83% of the patients were prescribed direct oral anticoagulants, 3% vitamin K antagonists, and 14% low-molecular-weight heparin (Table 2).

**Table 1:** Baseline characteristics

Parameter	Value (n = 29)
Female, n (%)	10 (34)
Age, years	66.6 ± 11.0
Age > 65 years, n (%)	16 (55)
Body mass index, kg/m^2^	32.4 ± 6.1
Body mass index ≥ 30 kg/m^2^, n (%)	19 (66)
Creatinine, mg/dl	1.0 (0.8–1.2)
Estimated glomerular filtration rate, ml/min/1.73 m^2^	68.8 ± 25.7
History of deep vein thrombosis, n (%)	8 (28)
History of PE, n (%)	4 (14)
History of chronic heart failure, n (%)	4 (14)
History of cancer, n (%)	4 (14)
Active cancer, n (%)	6 (21)
Major surgery within four weeks, n (%)	2 (7)
Contraindication to lytics, n (%)	3 (10)
Vasopressor required, n (%)	5 (17)
PESI score	121 ± 38
**Simplified PESI (sPESI) score**	2.3 ± 1.3
sPESI low risk (sPESI = 0), n (%)	1 (3)
sPESI high risk (sPESI ≥ 1), n (%)	28 (97)
**Concomitant deep vein thrombosis, n (%)**	24 (83)
Proximal (over the knee), n (%)	17 (59)
Distal (below knee), n (%)	15 (52)
Both (proximal & distal), n (%)	8 (28)
Bilateral, n (%)	5 (17)
**Biomarkers**	
Positive biomarkers^a^, n (%)	28 (97)
Hs-troponin T, pg/mL	80 (55–128)
NT-proBNP, pg/mL	2327 (1340–4166)
**PE risk stratification**	
High risk PE, n (%)	5 (17)
Intermediate-high PE, n (%)	22 (76)
Intermediate-low PE, n (%)	1 (3)
Low risk PE, n (%)	1 (3)

Data are presented as mean (standard deviations), median (interquartile ranges), or counts (percentages). ^a^Positive biomarkers include elevated NT-pro BNP (≥ 600 pg/mL) and/or troponin T (> 14 pg/mL)

Abbreviations: hs, high-sensitivity; NT-proBNP, N-terminal B-type natriuretic peptide; PE, pulmonary embolism; PESI, Pulmonary Embolism Severity Index; sPESI, simplified Pulmonary Embolism Severity Index

**Table 2:** Procedural characteristics

Parameter	Value (n = 29)
Time from diagnosis to procedure, h	21 (4–24)
Local anaesthesia, n (%)	29 (100)
Femoral access, n (%)	29 (100)
Contrast agent used, mL	115 (80–140)
Procedure time, min	68 (60–90)
Fluoroscopy time, min	15.3 (11.7–19.0)
**Anticoagulation before procedure**	
Unfractionated heparin, n (%)	29 (100)
**Anticoagulation at discharge**	
Low-molecular weight heparin, n (%)	4 (14)
Vitamin K antagonist, n (%)	1 (3)
Direct oral anticoagulant, n (%)	24 (83)
**Length of stay**	
Intensive care unit, days	2 (1–4)
In hospital stay, days	7 (6–11)

Data are presented as mean ± SD, median (interquartile ranges), or counts (percentages)

### Hemodynamic data

**Fig. 1:**
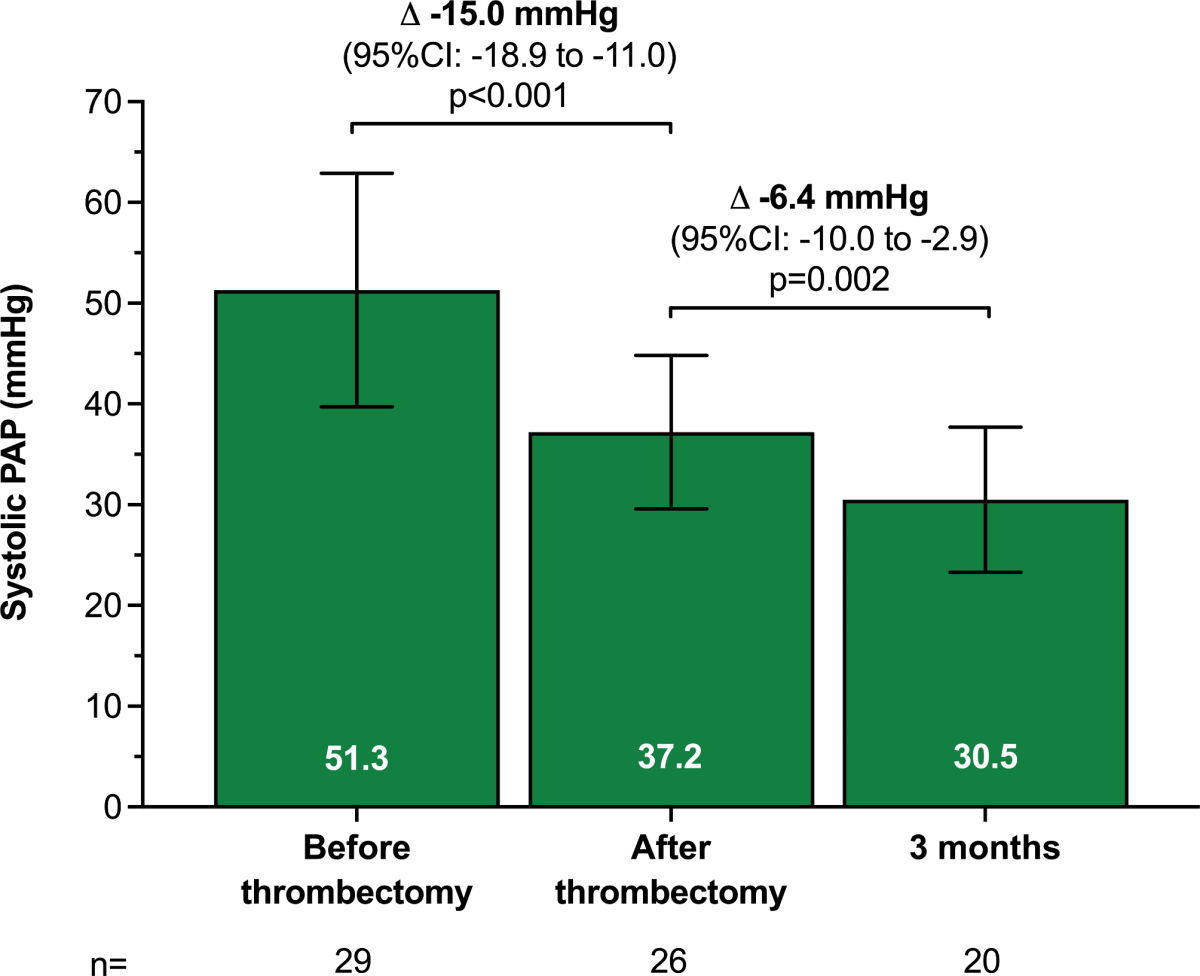
Systolic pulmonary artery pressure was assessed by right heart catheterization prior and after thrombectomy and at three months of follow-up. Data are means ± SD and mean change (95% CIs).

Table 3 summarizes the invasive and non-invasive hemodynamic data. Before thrombectomy, mean sPAP (51.3 ± 11.6 mmHg) and mPAP (29.8 ± 9.1 mmHg) were severely elevated. On table, mean sPAP, diastolic PAP (dPAP), and mPAP dropped by -15.0 mmHg (95%: -18.9 to -11.0; p < 0.001) (Fig. 1), -3.8 mmHg (95% CI: -6.6 to -1.1; p = 0.008), and − 8.4 mmHg (95% CI: -11.0 to -5.8; p < 0.001), respectively. While 2 (7%) patients had an sPAP > 70 mmHg before thrombectomy, none of the patients had an sPAP > 70 mmHg post-thrombectomy.

**Table 3:** Hemodynamic data

	Pre-procedure Mean ± SD	Post-procedure Mean ± SD	Mean change from pre-procedure^a^ Mean (95% CI)	p-value*	3 months Mean ± SD	Mean change between post-procedure and 3 months^b^ Mean (95% CI)	p-values^†^
Right heart catheterization							
Systolic PAP, mmHg	51.3 ± 11.6 n = 29	37.2 ± 7.6 n = 26	-15.0 (-18.9 to -11.0) n = 26	< 0.001	30.5 ± 7.2 n = 20	-6.4 (-10.0 to -2.9) n = 18	0.002
Diastolic PAP, mmHg	16.4 ± 8.2 n = 29	13.1 ± 5.0 n = 26	-3.8 (-6.6 to -1.1) n = 26	0.008	11.2 ± 5.1 n = 20	-2.9 (-6.3 to 0.4) n = 18	0.079
Mean PAP, mmHg	29.8 ± 9.1 n = 29	21.7 ± 6.4 n = 27	-8.4 (-11.0 to -5.8) n = 27	< 0.001	19.2 ± 5.6 n = 20	-2.7 (-5.7 to 0.3)	0.077
**Clinic BP**							
Systolic BP, mmHg	129 ± 22 n = 29	123 ± 16 n = 29	-6 (-15 to 3) n = 29	0.199	122 ± 13 n = 18	-2 (-12 to 9) n = 18	0.756
Diastolic BP, mmHg	79 ± 19 n = 29	70 ± 11 n = 29	-8.6 (-14 to -3) n = 29	0.005	74 ± 8 n = 18	-1 (-7 to 5)	0.748
Heart rate, b.p.m.	101 ± 20 n = 29	74 ± 10 n = 29	-27 (-35 to -20) n = 29	< 0.001	77 ± 11 n = 18	7 (1 to 14)	0.037
Peripheral oxygen saturation, %	93 ± 7 n = 29	97 ± 2 n = 29	4 (1 to 7) n = 29	0.006			
Supplemental oxygen, l/min	7 ± 5 n = 24	2 ± 2 n = 24	-5 (-6 to -3) n = 24	< 0.001			
Lactate, mmol/l	2.8 ± 3.0 n = 29	1.0 ± 0.5 n = 29	-1.8 (-2.9 to -0.7) n = 29	0.003			

Data are presented as means ± SD or counts (percentages). Before-after changes are summarized as means (95% confidence intervals [CI]). ^†^Matched data for change from before discharge to three months of follow-up.

^a^ Matched data for change from pre-procedure to post-procedure. ^b^ Matched data for change from post-procedure to three months of follow-up

Abbreviations: PAP, pulmonary artery pressure

**Fig. 2:**
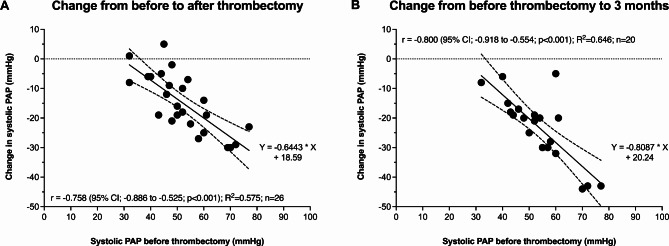
The scatter plot shows the correlation and the simple linear regression line (95% confidence interval) between systolic pulmonary artery pressure (PAP) before thrombectomy and its change on table (**A**) and at from before thrombectomy to 3 months (**B**). Pearson’s correlation coefficient was calculated for systolic PAP before thrombectomy and its change during follow-up

**Fig. 3:**
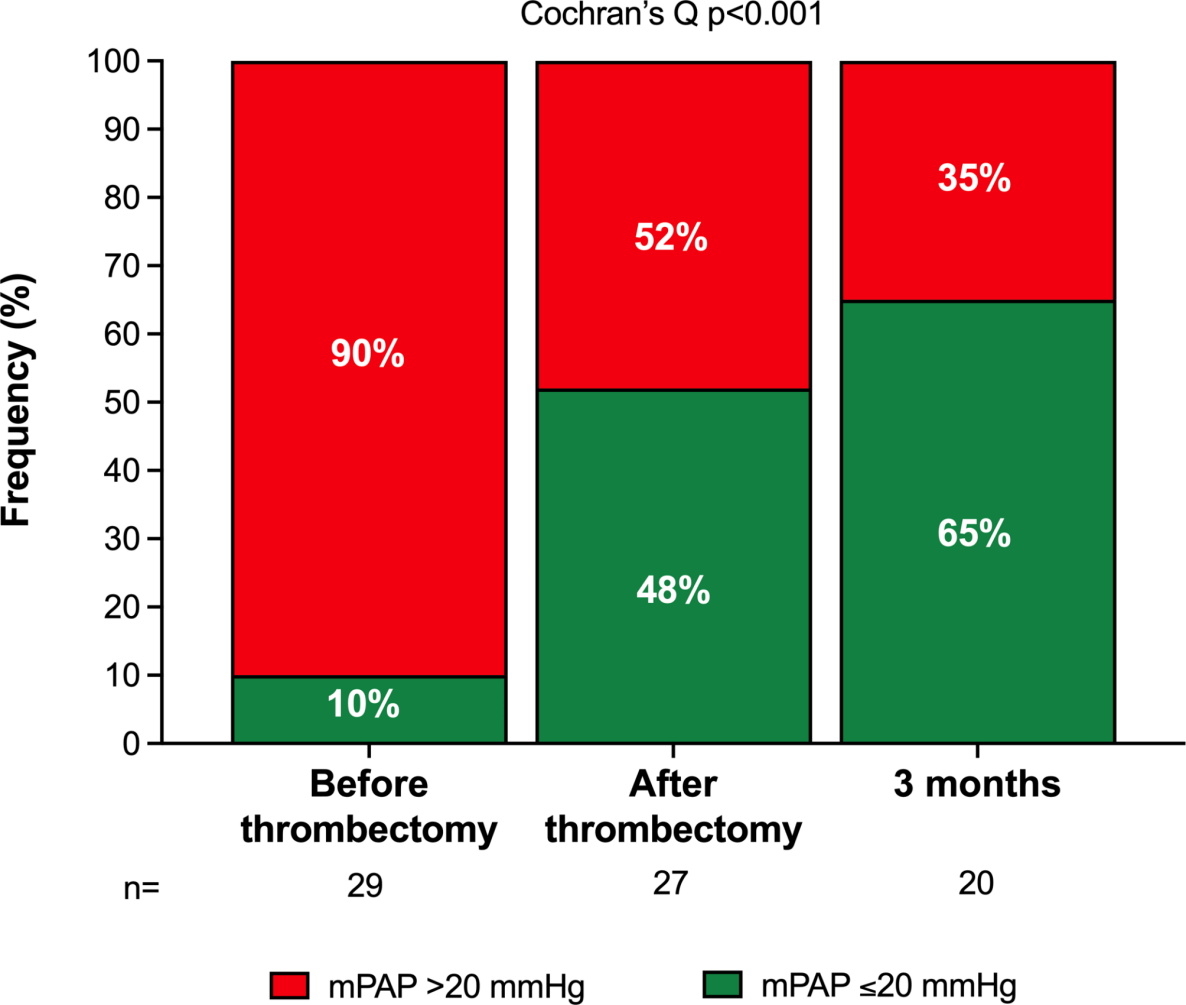
The figure shows the proportion of patients with mean pulmonary artery pressure (mPAP) > 20 mmHg and ≤ 20 mmHg measured using right heart catheterization immediately before and after thrombectomy and at three months of follow-up

From post-thrombectomy to three months, mean sPAP (p = 0.002) continued to decrease (Table 3). Compared with before thrombectomy, sPAP (-23.2 mmHg, 95% CI: -28.5 to -17.8; p < 0.001), dPAP (-6.7 mmHg, 95% CI: -11.6 to -1.8; p = 0.010), and mPAP (-12.0 mmHg, 95% CI: -16.8 to -7.2; p < 0.001) were significantly lower at three months. The change in sPAP from pre-thrombectomy to post-thrombectomy (r=-0.76; p < 0.001) and three months (r=-0.80; p < 0.001) strongly correlated with the pre-thrombectomy sPAP (Fig. 2). The proportion of patients with mPAP ≤ 20 mmHg increased on table and continued to increase until three months (Cochran’s Q p = 0.001) (Fig. 3).

Heart rate decreased significantly by -27 b.p.m. (95% CI: -35 to -20; p < 0.001) during the procedure, while systolic and diastolic blood pressure (BP) remained unchanged. Of note, patients in the lowest quartile of systolic BP before thrombectomy (mean systolic BP 104 ± 11 mmHg; range: 80 to 115) had an increase in systolic BP by 23 mmHg (95% CI: 14 to 31; p < 0.001) on table, whereas in those in the upper quartile (mean systolic BP 160 ± 5 mmHg; range: 155 to 177), systolic BP decreased by -18 mmHg (95% CI: -30 to -6; p = 0.008). Patients in the second and third quartiles did not have a change in systolic BP (both p > 0.189).

### Echocardiographic data

Before thrombectomy, the RV/LV ratio was increased (Table 4) and immediately reduced within two days by -0.37 (95% CI: -0.47 to -0.27; p < 0.001). TAPSE increased from 17.3 ± 6.4 mm pre-procedurally to 20.4 ± 4.0 mm before discharge (+ 3.0 mm; 95% CI: 0.5 to 5.5; p = 0.022). Improvements in right ventricular function were maintained at three months of follow-up. In line, the TAPSE/sPAP ratio, a parameter of RV arterial coupling, [14] tended to increase on table and significantly improved from discharge to three months (Table 4). The proportion of patients with impaired RV-arterial coupling, defined as TAPSE/sPAP ratio < 0.31 mm/mmHg, [14] decreased from 28% (5/18) at baseline to 0% before discharge and at three months (Cochran’s Q p = 0.007).

**Table 4:** Echocardiographic data

	Pre-procedure Mean ± SD	Before discharge* Mean ± SD	Mean change from baseline* Mean (95% CI)	p-value*	3 months^†^ Mean ± SD	Mean change between discharge and 3 months^†^ Mean (95% CI)	p-values^†^
LV ejection fraction, %	56.7 ± 8.5 n = 20	56.0 ± 5.8 n = 28	-1.6 (-6.7 to 3.4) n = 19	0.505	54.7 ± 10.1 n = 18	-1.8 (-7.4 to 3.7) n = 18	0.495
LV end-diastolic diameter, mm	41.9 ± 5.9 n = 26	49.0 ± 5.4 n = 28	7.4 (5.3 to 9.6) n = 25	< 0.001	48.6 ± 4.5 n = 18	-0.2 (-2.2 to 1.9) n = 18	0.866
RV end-diastolic diameter, mm	48.4 ± 6.9 n = 26	38.5 ± 7.2 n = 27	-8.9 (-12.3 to -5.6) n = 26	< 0.001	38.6 ± 4.6 n = 18	0.2 (-3.7 to 4.1) n = 17	0.924
RV/LV ratio	1.17 ± 0.22 n = 26	0.78 ± 0.13 n = 27	-0.37 (-0.47 to -0.27) n = 26	< 0.001	0.80 ± 0.11 n = 18	0.01 (-0.06 to 0.09) n = 17	0.718
TAPSE, mm	17.3 ± 6.4 n = 21	20.4 ± 4.0 n = 27	3.0 (0.5 to 5.5) n = 19	0.022	22.3 ± 2.9 n = 18	1.8 (-0.3 to 4.0) n = 18	0.093
sPAP, mmHg	37.7 ± 9.0 n = 19	31.9 ± 11.5 n = 24	-5.3 (-10.8 to -0.3) n = 19	0.063	25.1 ± 6.2 n = 15	-3.9 (-7.1 to -0.7) n = 14	0.020
TAPSE/sPAP, mm/mmHg	0.49 ± 0.20 n = 18	0.72 ± 0.27 n = 23	0.13 (-0.03 to 0.29) n = 15	0.102	0.94 ± 0.21 n = 15	0.16 (0.02 to 0.31) n = 14	0.031

Data are mean ± SD. Before-after changes are summarized as means (95% confidence intervals [CI]) or medians (95% CI). *Matched data for change from pre-procedure to before discharge. ^†^Matched data for change from before discharge to three months of follow-up

Abbreviations: LV, left ventricular; RV, right ventricular; sPAP, systolic pulmonary artery pressure; TAPSE, tricuspid annular plane excursion

### Clinical outcomes

The peripheral oxygen saturation increased from 93 ± 7% to 97 ± 2% (p = 0.006) from pre- to post-procedure, whereas the supplemental oxygen was reduced from 7 ± 5 l/min to 2 ± 2 l/min (p < 0.001). The arterial lactate level decreased (-1.8 mmol/l; 95% CI: -2.9 to -0.7; p = 0.003) on table. The perceived exertion, as measured using the original Borg CR10 scale, [15] improved on table (from a rating of 7 ± 2 to 3 ± 2; p < 0.001).

Median hs-troponin T decreased from 80 pg/ml (IQR: 55–128) to 25.5 pg/ml (IQR: 18–42) before discharge (median change: -37.5 pg/ml; 95% CI: -89 to -13; p < 0.001) and to 11 pg/ml (IQR: 8–27) at three months (median change from pre-thrombectomy: -58 pg/ml; 95% CI: -131 to -42; p = 0.002). Before thrombectomy, hs-troponin T was increased (> 14 pg/ml) in 97% (28/29) of the patients. Following thrombectomy, the proportion of patients with increased hs-troponin T decreased from 90% (20/22) before discharge to 38% (5/13) at three months (Cochran’s Q for change from baseline to three months p = 0.006).

Likewise, NT-proBNP substantially decreased from 2327 pg/ml (IQR: 1341–4167) to 344 pg/ml (IQR: 140–716) before discharge (median change: -2172 pg/ml; 95% CI: -3661 to -1328; p < 0.001) and to 166 pg/ml (IQR: 82–260) at three months (median change from pre-thrombectomy: -2226.5 pg/ml; 95% CI: -3904 to -1693; p < 0.001).

### Safety outcomes

There were no procedure-related major adverse events between baseline to 48 h and survival from baseline to 30 days of follow-up. Importantly, no patient died during 30 days of follow-up.

## Discussion

This study assessed whether the hemodynamic effects of mechanical thrombectomy using the FlowTriever System are maintained through three months post-procedure in acute PE with right heart overload. The key findings are: (i) mechanical thrombectomy for acute PE was safe, (ii) immediately reduced PAP, improved right heart function and clinical symptoms, and (iii) mean sPAP continued to decrease through three months of follow-up.

The 2019 ESC Guidelines for the Diagnosis and Management of Acute PE recommend catheter-directed treatment for reperfusion in patients with high-risk PE, in case systemic thrombolysis fails or is contraindicated [3, 7]. The feasibility and safety of mechanical thrombectomy using the FlowTriever System in patients with intermediate and high-risk PE were recently investigated in the multicenter, single-arm FLARE study (n = 104 patients) and the prospective FlowTriever All-Comer Registry for Patient Safety and Hemodynamics (FLASH) (n = 1000 patients). In line with the findings presented herein, in FLARE study [11] and FLASH, the mean RV/LV ratio was immediately reduced by 0.38 and 0.25, respectively [12]. Thrombectomy was also associated with reductions in sPAP and mPAP [11, 12] but the degree of mPAP changes varied across studies (-7.6 mmHg in FLASH [12] versus − 2.0 mmHg in FLARE) [11]. In the present study, mPAP was reduced by -8.4 mmHg. Interestingly, a progressive decline in sPAP was observed through three months of follow-up. This sustained reduction in sPAP could help to prevent the development of post-PE syndrome (PPES), which is defined as new or progressive dyspnea, exercise intolerance, and/or impaired functional or mental status after at least three months of adequate anticoagulation following acute PE, reported in up to 40–60% of PE survivors [16].

Compared with baseline, where 90% of the patients had pulmonary hypertension as defined by mPAP > 20 mmHg, about 35% had sustained mPAP > 20 mmHg at three months. This is of relevance since mPAP elevations > 20 mmHg predict mortality [17–20]. Therefore, mechanical thrombectomy may improve acute outcomes by disrupting the shock spiral and mortality by reducing pulmonary arterial pressures in the long term. This is supported by the observation that immediately following the procedure (i) oxygen saturation increased while oxygen supply was reduced (-5 l O_2_ l /min), (ii) arterial lactate level decreased, (iii) heart rate was lowered by 27 bpm, and (iv) hs-troponin T levels normalised. However, mPAP remains elevated in about a third of the patients. This group may include patients with previously undiagnosed pulmonary hypertension and those at increased risk for chronic thromboembolic pulmonary hypertension (CTEPH), requiring further follow-up.

Further, mechanical thrombectomy improved RV function and RV-arterial coupling, assessed using the TAPSE/sPAP ratio. The ratio of TAPSE/sPAP, where TAPSE estimates contractility and sPAP reflects afterload, represents a non-invasive surrogate of end-systolic/arterial elastance (Ees/Ea) ratio and associates with functional class and prognosis in pulmonary hypertension [14, 21, 22].

None of the patients included in this study died within 30 days post-thrombectomy. In FLASH, all-cause mortality at 30 days was 0.8%, [12] which is lower than the historical 30-day mortality rate for intermediate-risk PE patients receiving anticoagulation only (up to 10%) [23]. In the absence of evidence from prospective, randomised, controlled trials assessing cardiovascular outcomes, a retrospective analysis comparing patients with intermediate-high and high-risk PE who received mechanical thrombectomy (n = 28) or routine care (n = 30) suggested that mechanical thrombectomy might indeed improve in-hospital mortality (3.6% vs. 23.3%, p < 0.05) [24].

In contrast to systemic thrombolysis, recommended in high-risk PE but associated with increased bleeding risk, particularly in elderly patients, [4] we observed no major adverse events. There were also no major procedure- or device-related adverse events documented.

This study adds to previous single-arm studies and registries showing that mechanical thrombectomy using large-bore thrombus aspiration is feasible and safe, reduces thrombus burden, and immediately improves hemodynamics in intermediate and high-risk PE [11, 12]. Moreover, this is the first study indicating that the improvement in invasive hemodynamic parameters was maintained at three months of follow-up. Therefore, if mechanical thrombectomy improves cardiovascular outcomes and reduced morbidity and mortality in randomized controlled trials, the procedure offers an alternative approach to systemic thrombolysis for treating acute PE with right heart overload, especially in patients with contraindications for or hemodynamically stable patients who do not benefit from systemic thrombolysis.

### Limitations

Several limitations of this analysis need to be acknowledged. Because of the non-randomized study design and the lack of a comparator treatment group, we cannot exclude selection bias and unspecific treatment effects. Therefore, the data should be interpreted with caution and as hypothesis-generating. This feasibility study’s size was small and not based on an *a priori* power calculation to assess hemodynamic effects. All procedures herein were performed using the FlowTriever System, so the findings cannot be extrapolated to other catheter systems. The procedures were performed by only two experienced operators trained in structural interventions, which may explain the low rate of adverse events observed herein.

## Conclusion

Mechanical thrombectomy for acute intermediate and high-risk PE with RV overload was safe and immediately reduced PAP, improved right heart function and RV-arterial coupling. Moreover, the reduction in PAP was maintained for three months. Randomized, controlled trials are required to finally assess the role of mechanical thrombectomy in the management of acute PE.

## Data Availability

The datasets used and analyzed during the current study are available from the corresponding author on reasonable request.

## Abbreviations and acronyms

dPAP: diastolic pulmonary artery pressure
ESC: European Society of Cardiology
Hs: high sensitivity
ICU: intensive care unit
LV: left ventricle
mPAP: mean pulmonary artery pressure
NT-proBNP: N-terminal B-type natriuretic peptide
PAP: pulmonary artery pressure
PE: pulmonary embolism
RV: right ventricle
sPAP: systolic pulmonary artery pressure
TAPSE: tricuspid annular plane systolic excursion
